# Spectral and cepstral measurements in women with behavioral dysphonia

**DOI:** 10.1590/2317-1782/20232022327en

**Published:** 2023-11-10

**Authors:** Gabriela Marques Paiva, Priscila Oliveira Costa Silva, Layla Jamilly Andrade da Silva, Kézia Alves Nascimento, Ana Beatriz da Veiga e Silva, Samuel Ribeiro de Abreu, Anna Alice Figueiredo de Almeida, Leonardo Wanderley Lopes

**Affiliations:** 1 Universidade Federal da Paraíba - UFPB - João Pessoa (PB), Brasil.

**Keywords:** Voice, Voice Disorders, Voice Quality, Acoustics

## Abstract

**Purpose:**

To investigate whether there are differences in cepstral and spectral acoustic measures between women with behavioral dysphonia with and without laryngeal lesions and verify whether there is a correlation between such measures and the auditory-perceptual evaluation of voice quality.

**Methods:**

The sample comprised 78 women with behavioral dysphonia without laryngeal lesions (BDWOL) and 68 with behavioral dysphonia with laryngeal lesions (vocal nodules) (BDWL). Cepstral peak prominence (CPP), cepstral peak prominence-smoothed (CPPS), spectral decrease, and H1-H2 (difference between the amplitude of the first and second harmonics) were extracted. They were submitted to the auditory-perceptual evaluation (APE) of the grade of hoarseness (GH), roughness (RO), breathiness (BR), and strain (ST).

**Results:**

BDWL women had higher H1-H2 values and lower CPP and CPPS values than BDWOL women. More deviant voices had lower CPP and CPPS values. Breathy voices had lower CPP and CPPS values and higher H1-H2 values than rough ones. There was a weak negative correlation between CPP and RO, a moderate negative correlation with GH, and a strong negative correlation with BR. CPPS had a moderate negative correlation with GH, RO, and BR. H1-H2 had a weak positive correlation with BR. There was a weak positive correlation between spectral decrease and ST.

**Conclusion:**

H1-H2, CPP, and CPPS were different between BDWOL and BDWL women. Furthermore, cepstral and spectral measures were correlated with the different APE parameters.

## INTRODUCTION

Acoustic analysis is a tool capable of characterizing the vocal signal both quantitatively and qualitatively, thus providing estimates of physiological aspects underlying voice production^(1)^. From the clinical perspective, each acoustic measure is expected to be sensitive to variations in voice production aerodynamics and biomechanics and auditory-perceptual evaluation (APE) parameters.

There is a greater amount of research and applications using acoustic measures based on statistics and short-term perturbations in oscillation frequency (f0), short-term perturbations in amplitude, and perturbations in waveform^(2)^. However, there are important restrictions to these measures’ reliability and reproducibility, especially in the assessment of more intensely deviant voices^(2)^. In this regard, recent research^(3,4)^ and consensus^(5)^ support the use of spectral measures based on either Fourier transform/LPC spectrum (linear predictive coding) or the cepstrum to acoustically assess dysphonic voices. Among these, the cepstral peak prominence (CPP) and cepstral peak prominence-smoothed (CPPS) have been indicated as reliable measures to assess dysphonic voices, regardless of the intensity of vocal deviation^(1,3-6)^.

CPP and CPPS demonstrate to what extent f0 harmonics are individualized and stand out from the noise level in the signal^(3)^. Signals with greater regularity and less noise have greater definition and amplitude in the dominant cepstral peak. CPP and CPPS have two main advantages in relation to traditional perturbation and noise measures: they do not depend on f0 estimates to be obtained and can be extracted from both sustained vowels and linked speech. Moreover, CPPS values have been able to discriminate Brazilian Portuguese speakers with and without vocal deviation; distinguish predominantly rough, breathy, or strained voices; and detect a strong negative correlation with the grade of hoarseness (GH) and breathiness (BR), a moderate negative correlation with roughness (RO, and a weak negative correlation with strain (ST)^(6)^.

In the field of spectral measures, those related to spectral decrease (which include parameters that reflect the harmonic energy spectrum, comparing differences in decibels [dB] between relative amplitudes of two harmonics) are among the main predictors of vocal hyperfunctioning or the breathiness component related to voice production^(7,8)^. The difference in amplitude between the first and second harmonics (H1-H2) and the characterization of the spectral decrease in dBs are among the main measures recommended for use in clinical practice^(7)^. A systematic review with meta-analysis reported that H1-H2 was among the main measures to predict the presence and intensity of breathiness in voice emission^(4)^.

H1-H2 is a measure related to the spectral slope, glottal airflow pulse slope, vocal fold opening quotient, the thickness of the free edges of the vocal folds, and vibration asymmetry between vocal folds^(9,10)^. It is also associated with breathiness and strain components. H1-H2 can furnish insights into physiological mechanisms underlying voice production and is potentially useful in the clinical assessment of dysphonic individuals.

Patients with phonotraumatic vocal fold lesions generally have higher H1-H2 values than vocally healthy individuals^(8)^. Higher H1-H2 values are associated with less contact between vocal folds, vibration pattern with less abrupt closure between vocal folds, longer open phase of the glottal cycles, greater constriction/narrowing of the vocal tract, and breathy voice quality^(8,9,11,12)^. On the other hand, lower H1-H2 values are compatible with greater glottal closure or abrupt glottal closure of the vocal folds, with greater phonatory strain in the voice quality^(8)^, greater longitudinal (anteroposterior) strain of the vocal folds^(13)^, and greater vertical thickness of the free edge of the vocal folds^(13)^.

H1-H2 varies greatly in intersubject comparison^(10)^. Hence, it must be investigated in different laryngeal conditions and voice quality deviations to understand its usefulness in a clinical context. Moreover, CPP/CPPS, H1-H2, and spectral decrease have been considered complementary measures to assess vocal deviation and, therefore, estimate structural and kinematic changes in the vocal folds underlying the voice production process^(14,15)^.

Behavioral dysphonia corresponds to 65% of the cases found in voice clinical practice^(16)^, and 10 to 40% of these cases can occur without structural changes in the larynx^(17)^. Patients with behavioral dysphonia, regardless of having phonotraumatic lesions, have greater muscle activity and excessive effort or strain in the intrinsic and/or extrinsic musculature of the larynx during voice production^(18)^. In their turn, tissue lesions associated with phonotrauma, such as nodules, polyps, and edema in the vocal folds, have the potential to change the vibration and kinematic characteristics of voice production^(18)^. Given the prevalence of these conditions, it is important to identify measures capable of characterizing and monitoring these patients’ voice production with a potential application in clinical practice.

Thus, considering the relevance of these measures in clinical voice assessment and the scarcity of studies in Brazilian Portuguese speakers, this research aimed to investigate whether there are differences in cepstral (CPP and CPPS) and spectral (H1-H2 and spectral decrease) acoustic measures between women with behavioral dysphonia with (BDWL) and without laryngeal lesions (BDWOL) and whether there is a correlation between these measures and APE of voice quality.

## METHODS

### Study design

This cross-sectional descriptive study was appraised and approved by the originating institution’s Research Ethics Committee under evaluation report no. 2.677.777/2018. All participants signed an informed consent form, agreeing to participate in the research.

### Sample

The research sample comprised 146 dysphonic women with a mean age of 33.10±11.26 years, assessed at the originating institution’s voice laboratory before voice therapy. Among them, 78 were diagnosed with behavioral dysphonia with no laryngeal lesion, and 68 were diagnosed with behavioral dysphonia with laryngeal lesions (vocal nodules).

The eligibility criteria to select participants were as follows: being females, as the acoustic measures investigated in this study are influenced by sex^(19)^; being 18 years or older to avoid the period of voice changes; having voice complaints, based on the affirmative answer to the question, “Do you currently perceive any problem in your voice?”; being diagnosed with behavioral dysphonia (with vocal nodules or without laryngeal lesions), confirmed with visual laryngeal examination by an otorhinolaryngologist.

The participant exclusion criteria were occupational voice users or women who had been previously submitted to head and neck surgery (including cases of laryngeal microsurgery) or voice therapy. Other exclusion criteria were related to the quality of the signals of the collected voices - those whose voice signals had a signal-to-noise ratio (SNR) below 30 dBSPL were excluded^(5)^.

**Figure 1:**
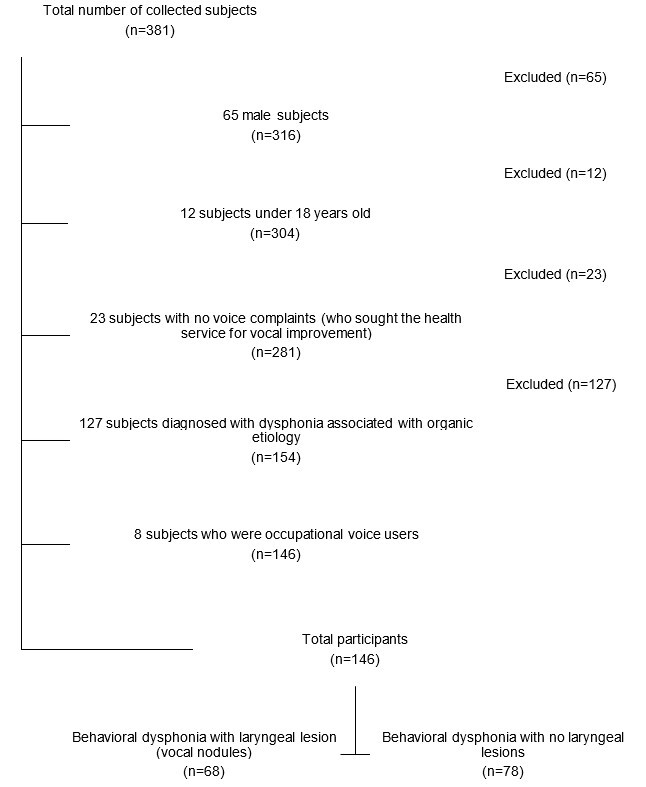
Flowchart of research participants, according to eligibility criteria

Figure 1 shows information on sample selection based on inclusion and exclusion criteria.

Participants were allocated to one of the following groups:

- Behavioral dysphonia without laryngeal lesions (BDWOL) - including participants whose dysphonia etiology was associated with vocal behavior, with self-reported voice problems. None of this group’s participants had structural changes in the larynx; 25 of them had an otorhinolaryngological diagnosis of mid-posterior glottic chink, 11 participants were diagnosed with supraglottal constriction, and 42 participants had a normal larynx.
- Behavioral dysphonia with laryngeal lesions (BDWL) - women with structural changes in the vocal folds, whose dysphonia etiology is associated with vocal behavior, with self-reported voice problems. All 68 participants in this group had an otorhinolaryngological diagnosis of vocal nodules and glottic chink. This research approached the diagnosis of vocal nodules because this is the main laryngeal lesion associated with behavioral dysphonia^(^ 16 ^)^.

### Data collection procedure

Data in this research were collected at the voice laboratory of a higher education institution. Participants were initially assessed with a form approaching personal data and voice complaints. Then, their voice samples were recorded.

Their voices were collected with Fonoview software, version 4.5, by CTS Informática, using an all-in-one Dell desktop, a Sennheiser unidirectional cardioid microphone, manufactured, model E-835, placed on a pedestal and attached to a Behringer preamplifier, model U-Phoria UMC 204. The voices were collected in an acoustically treated recording booth, with noise under 50 dB SPL, 44000-Hz sampling rate, 16 bits per sample, and the microphone 10 cm away from the participant’s mouth.

Participants were standing to collect their voices, with the pedestal in front of them, keeping their mouths at the set distance from the microphone. They were instructed on the collection procedures before recording their voices. They were asked to emit the sustained vowel /a/ for at least 5 seconds and count from 1 to 10 in self-reported habitual frequency and intensity.

During voice recording, the signals were visually monitored in the Fonoview display, observing signal duration (lasting at least 5 s) and the presence of peak clipping. Hence, if the emission lasted less than 5 s or had peak clipping, the participant was asked to rerecord to obtain the established criteria.

After the session of data collection and voice sample recording, the participants were referred for otorhinolaryngological examinations, including visual laryngeal examination with telelaryngoscopy using a rigid endoscope. All examinations were performed at the same reference service, and participants received a written report with the laryngeal diagnosis. Examination results were used as criteria to select research participants.

Then, before proceeding with APE and taking acoustic measures, the voice signals’ SNR was obtained in the VoxMore script of Praat open-access software (Paul Boersma and David Weenink, University of Amsterdam, the Netherlands), version 5.3.84. All samples of the 146 selected participants met the pre-established signal quality criteria^(5)^, with SNR at 45.37± 3.1 dB SPL.

The vowel and counting samples were presented to speech-language-hearing therapists who specialized in voice with more than 20 years of experience in assessing and treating voice disorders. The APE session took place in a silent room, with earphones attached to a laptop computer, at a self-reported comfortable volume to the evaluator. In this evaluation, the speech-language-hearing therapist listened to the voice samples (counting and sustained vowels) in sequence, with a 1-second interval in between them. Then, they judged GH, RO, BR, and ST with markings on a 0-to-100-mm visual analog scale (VAS). Markings closer to 0 indicated less voice quality deviation, and those closer to 100 indicated greater voice quality deviation^(20)^.

At the end of the APE session, 20% (n = 30) of the voice samples were randomly repeated to analyze intrarater reliability with Cohen’s kappa coefficient. The judge’s kappa coefficient was 0.83, indicating good agreement.

VAS cutoff scores^(20)^ were used to classify the voices regarding vocal deviation and GH. Signals that scored ≤ 35.5 mm were considered as having adapted voice quality or normal voice quality variability, whereas voices with values > 35.5 mm were classified as deviant. Then, VAS was corresponded with a numerical scale, as follows: degree 1 (0 - 35.5 mm) was related to normal voice quality variability; degree 2 (35.6 - 50.5 mm), with mild to moderate deviation; degree 3 (50.6 - 90.5 mm), with moderate deviation; and degree 4 (90.6 - 100 mm), with intense deviation^(20)^. After the APE result, 11 women were found to have adapted voice quality, 74 had mild to moderate vocal deviation, and 61 had moderate vocal deviation. None of the participants selected for this study had intense vocal deviation. Higher RO, BR, and ST values in VAS of the 74 women with vocal deviation were used to classify the predominant voice quality deviation as roughness, breathiness, or strain.

The 11 participants with adapted voice quality were maintained in the study, considering that the diagnostic confirmation of dysphonia is multidimensional and that behavioral conditions may have multiple interactions between physiological, acoustic, auditory-perceptual, and self-perceptive parameters of the voice problem^(18)^. Thus, women with behavioral dysphonia may have adapted voice quality, despite reporting voice complaints/symptoms and structural changes in the larynx. Eight out of the 11 women in this sample with adapted voice quality were in the BDWOL group, while only three were allocated to the BDWL group.

Counting from 1 to 10 (linked speech) was the only speech task used in the reference study^(20)^ addressing the Brazilian reality to determine VAS cutoff scores. Even though this may be a limitation of this study, it used the cutoff scores proposed by Yamasaki et al.^(20)^ because it approaches only the four internationally considered degrees of deviation (adapted or normal voice quality variability, mild to moderate, moderate, and intense) and is the main Brazilian cutoff reference for this classification. Moreover, the task of counting from 1 to 10 was included (along with the sustained vowel) in the APE.

Then, the CPP, CPPS, spectral decrease, and H1-H2 acoustic measures were taken from the sustained vowel /a/ samples. The amplitude of the first and second harmonics is influenced by the type of vowel collected, especially regarding timbre (open vs. close)^(21)^. Hence, vowel /a/ is the most reliable one to take H1-H2 measures because the frequency of the first formant is high enough not to interfere with the harmonics at the lower frequency bands. Moreover, the first and second harmonics are more distinct in this vowel^(8,21)^.

The measures were taken automatically with the VoxMore script^(22)^ in Praat (v.5.3.84). This script takes measures from the 3 central seconds of the sustained vowel. Concerning H1-H2, it takes measures with the discrete Fourier transform, using 50-ms windows. H1-H2 for each window was defined as the difference in dB between the amplitudes of the first and second harmonics in the frequency spectrum^(8)^. CPP was extracted with 41-ms windows, and the final CPP value for each sample corresponded to the mean of all 3-second-interval analysis frames^(23,24)^. CPPS was obtained in 2-ms windows, and its final value corresponded to the mean of all 3-second-interval analysis frames^(3,25)^.

### Data analysis

All independent variables addressed in the study were submitted to descriptive statistical analysis, including the means and standard deviations. The Shapiro-Wilk test was initially used to verify the normality distribution curve of the variables investigated (GH, RO, BR, ST, CPP, CPPS, spectral decrease, and H1-H2) in the study sample. Thus, given the normal distribution of all these variables, the independent samples t-test was used to compare the means of the acoustic measures and APE between the BDWOL and BDWL groups. The one-way ANOVA test was used to compare acoustic measures between the groups regarding GH and the predominant voice quality, followed by the Tukey post-test when there was a statistically significant difference between the groups in ANOVA. The Pearson correlation test was used to verify whether there was a correlation between values of acoustic measures and APE parameters (GH, RO, BR, and ST).

Correlation coefficients were used to assess and quantify the degree of linear relationship between acoustic (CPP, CPPS, spectral decrease, and H1-H2) and APE variables (GH, RO, BR, and ST), observing whether they changed together and to what degree. This research classified correlation coefficients as follows: 0.1 to 0.3 indicated a weak correlation; 0.4 to 0.6 indicated a moderate correlation; and above 0.6 indicated a strong correlation.

All analyses were performed in the Statistical Package for the Social Sciences (SPSS), version 2.0. The level of significance was set at 5%.

## RESULTS

Differences were found in all H1-H2, CPP, and CPPS acoustic measures between the participating groups (Table 1). BDWL women had higher H1-H2 values (p = 0.024) and lower CPP (p = 0.017) and CPPS values (p = 0.004) than BDWOL women. There was no difference in spectral decrease values between BDWL and BDWOL (p = 0.504).

**Table 1:** Comparison of mean acoustic measures and auditory-perceptual evaluation parameters between women with behavioral dysphonia, with and without laryngeal lesions

**MEASURES**	**GROUP**		**p-value**
	**BDWL**	**BDWOL**	
	**Mean and SD**	**Mean and SD**	
**GH**	43.03±8.18	55.83±12.10	<0.001*
**RO**	38.24±11.12	51.38±12.79	<0.001*
**BR**	38.01±13.66	48.19±17.39	<0.001*
**ST**	28.62±11.10	36.22±13.73	<0.001*
**CPP**	27.52±3.60	25.29±5.45	0.017*
**CPPS**	16.36±2.39	14.77±3.10	0.004*
**Spectral decrease**	-14.68±0.52	-15.33±0.63	0.504
**H1-H2**	1.65±5.31	3.64±5.16	0.024*

Independent samples t-test

* Significant values - p-value < 0.05

Caption: **SD** = standard deviation; **GH** = Grade of hoarseness of the voice; **RO** = Roughness; **BR** = Breathiness; **ST** = Strain; **H1-H2** = difference in amplitude between the first two harmonics of the voice spectrum; **CPP** = cepstral peak prominence; **CPPS** = cepstral peak prominence-smoothed; **BDWL** = behavioral dysphonia with laryngeal lesion; **BDWOL** = behavioral dysphonia without laryngeal lesion

There were differences in CPP (p < 0.001) and CPPS values (p < 0.001) in relation to GH (Table 2). The Tukey post-test compared the groups pair by pair and found differences in CPP values in the comparison between adapted x mild to moderate (p = 0.004), adapted x moderate (p < 0.001), and mild to moderate x moderate (p < 0.001). Likewise, CPPS values were different in the comparison between adapted x mild to moderate (p = 0.005), adapted x moderate (p < 0.001), and mild to moderate x moderate (p < 0.001). In general, more deviant voices had lower CPP and CPPS values. There were no differences in spectral decrease (p = 0.220) and H1-H2 values (p = 0.504) in relation to GH (p = 0.504).

**Table 2:** Comparison of mean acoustic measures and auditory-perceptual evaluation parameters between women with behavioral dysphonia with and without laryngeal lesions, regarding the grade of hoarseness in their voices

**MEASURES**	**GRADE OF HOARSENESS**			**p-value**
	**Adapted**	**Mild to moderate**	**Moderate**	
	**Mean and SD**	**Mean and SD**	**Mean and SD**	
**CPP**	31.86±3.25	27.52±3.39	24.25±5.04	<0.001*
**CPPS**	18.98±2.22	16.33±2.32	14.15±2.71	<0.001*
**Spectral decrease**	-13.47±1.04	-15.23±0.57	-14.96±0.66	0.220
**H1-H2**	3.76±2.91	1.54±4.89	3.62±5.92	0.057

One-way ANOVA

* Significant values - p-value < 0.05

Caption: **SD** = standard deviation; **H1-H2** = difference in amplitude between the first two harmonics of the voice spectrum; **CPP** = cepstral peak prominence; **CPPS** = cepstral peak prominence smoothed

There were differences in CPP (p < 0.001), CPPS (p < 0.001), and H1-H2 measures (p = 0.022) in relation to the predominant voice quality (Table 3). The Tukey post-test compared the groups pair by pair and found differences in CPP (p < 0.001), CPPS (p < 0.001), and H1-H2 values (p = 0.018) only in the comparison between rough and breathy voices. The latter had lower CPP and CPPS values and higher H1-H2 values than rough voices. There were no differences in spectral decrease values (p = 0.671) in relation to the predominant voice quality.

**Table 3:** Comparison of mean acoustic measures and auditory-perceptual evaluation parameters between women with behavioral dysphonia with and without laryngeal lesions, regarding their predominant voice quality.

**MEASURES**	**PREDOMINANT VOICE QUALITY**			**p-value**
	**Rough**	**Breathy**	**Strained**	
	**Mean and SD**	**Mean and SD**	**Mean and SD**	
**CPP**	27.53±4.13	24.61±4.49	28.38±1.58	<0.001*
**CPPS**	16.14±2.45	14.57±2.78	16.80±1.47	<0.001*
**Spectral decrease**	-15.49±0.64	-15.30±0.54	-5.84±2.75	0.671
**H1-H2**	0.98±5.44	3.68±5.23	4.35±5.14	0.022*

One-way ANOVA test

* Significant values - p-value < 0.05

Caption: **SD** = standard deviation; **H1-H2** = difference in amplitude between the first two harmonics of the voice spectrum; **CPP** = cepstral peak prominence; **CPPS** = cepstral peak prominence smoothed

Lastly, CPP had a weak negative correlation with RO (< 0.001), a moderate negative correlation with GH (< 0.001), and a strong negative correlation with BR (< 0.001) (Table 4). CPPS had a moderate negative correlation with GH (< 0.001), RO (< 0.001), and BR (< 0.001). H1-H2 had only a weak positive correlation with BR (p = 0.013). There was a weak positive correlation between spectral decrease and ST (p = 0.003).

**Table 4:** Correlation between the intensity of vocal deviation in the various auditory-perceptual parameters and the acoustic measures

**ACOUSTIC MEASURE**	**VARIABLES**							
	**GH**		**RO**		**BR**		**ST**	
	**Corr**	**p-value**	**Corr**	**p-value**	**Corr**	**p-value**	**Corr**	**p-value**
**CPP**	-0.45	<0.001*	-0.31	<0.001*	-0.60	<0.001*	-0.03	0.693
**CPPS**	-0.52	<0.001*	-0.40	<0.001*	-0.58	<0.001*	-0.10	0.210
**Spectral decrease**	0.07	0.933	-0.021	0.802	-0.086	0.303	0.24	0.003*
**H1-H2**	-0.12	0.208	-0.15	0.783	0.08	0.013*	-0.08	0.313

Pearson correlation test

* Significant values (p ≤ 0.05)

Caption: **GH** = Grade of hoarseness of the voice; **RO** = Roughness; **BR** = Breathiness; **ST** = Strain; **Corr** = correlation coefficient; **H1-H2** = difference in amplitude between the first two harmonics of the voice spectrum; **CPP** = cepstral peak prominence; **CPPS** = cepstral peak prominence smoothed

## DISCUSSION

Spectral/cepstral acoustic measures have stood out as the most promising ones to assess and monitor dysphonic voices. They have proved to be sensitive to different APE components (such as GH, BR, and ST)^(6)^ and kinematic and vibration characteristics associated with normal and dysphonic voice production^(14)^.

BDWL women in this research had higher H1-H2 values and lower CPP and CPPS values than BDWOL women. There is no H1-H2 normative data for Brazilian Portuguese-speaking women, whereas for English-speaking ones there they range from 3.3 to 8.4 dB^(21)^. BDWOL and BDWL women in this study respectively obtained 1.65±5.31 and 3.64±5.16. These values may suggest greater glottal closure or abrupt glottal closure of the vocal folds, with greater phonatory strain in the voice quality^(8)^, increased longitudinal (anteroposterior) strain of the vocal folds^(13)^, and increased vertical thickness of the free edge of the vocal folds^(13)^ in dysphonic women.

As for H1-H2, the higher values in BDWL than in BDWOL women may indicate less contact between vocal folds, a vibration pattern with less abrupt closure between vocal folds, longer open phase of the glottal cycles, greater constriction/narrowing of the vocal tract, and greater breathiness^(8,9,11,12)^.

BDWL patients tend to have a glottic chink, which may explain the greater breathiness and higher H1-H2 values^(8,26,27)^. The BDWOL group probably had a hyperfunctional component and greater glottal closure or abrupt glottal closure, justifying the lower H1-H2 values.

Previous studies^(8,9,26)^ compared patients with phonotraumatic lesions and vocally healthy individuals, observing that H1-H2 values were lower in the group with lesions. However, data in the present research indicated higher H1-H2 values in BDWL than in BDWOL women. Thus, there seems to be a continuum in H1-H2 measures, with higher values in vocally healthy people, decreased H1-H2 in cases of BDWL, and lower values in BDWOL. This finding may be compatible with more intense glottal closure in people with behavioral dysphonia than in vocally healthy ones, reflected in lower H1-H2 values.

There is also a moderate positive correlation between vibration asymmetry in the vocal folds and H1-H2 values^(28)^. Hence, the vocal nodules in the BDWL group may cause greater vibration asymmetry between vocal folds and explain the higher H1-H2 values in this group.

As for the comparison between acoustic measures and GH, only CPP and CPPS had differences, with lower values in patients with more deviant voices. CPP and CPPS have been recommended as useful measures to monitor voice quality deviation during the patient’s treatment^(3,6)^. These measures are sensitive to the periodicity of the signal and amplitude of the harmonic energy and are, therefore, recommended as the main measure to monitor GH^(5)^.

The literature presents conflicting H1-H2 results in relation to GH. No consistent H1-H2 changes were found in the comparison between voices with greater or less GH^(29)^. Voices with intense deviations have values similar to those of voices with no deviations^(29)^. Furthermore, H1-H2 values did not decrease after voice therapy in dysphonic patients, despite the decreased GH and improved videostroboscopy laryngeal parameters^(30)^. Hence, H1-H2 seems to complement other measures like CPP and CPPS, being useful in understanding the predominant voice quality and the physiology underlying the voice production process.

Dysphonic women with predominantly breathy voices had lower CPP and CPPS values and higher H1-H2 values than dysphonic women with predominantly rough voices. It is challenging to establish the difference between dysphonic rough and breathy voices based on acoustic measures because these two components overlap in the same deviant vocal signal.

There are three acoustic indicators of the presence of breathiness in voice production: signal periodicity and first harmonic spectral decrease and amplitude^(24)^. Signal periodicity can explain 80% of the variable perception of breathiness degree in a voice^(24)^. CPP and CPPS are related to signal periodicity, which justifies the differences found in these measures between rough and breathy voices in this research, with lower values in breathy voices.

Breathy voices tend to have greater amplitude in the first harmonic, with relatively weak higher harmonics, which justifies the higher H1-H2 values in these than in rough voices^(24)^. Since these three measures are considered sensitive to the presence of breathiness in voice production^(31)^, they help understand the difference in values between predominantly rough and breathy voices.

In this research, GH had a moderate negative correlation with CPP and CPPS. This finding supports the current indication of CPP and CPPS as the main measures to identify the presence and intensity of vocal deviation in dysphonic voices^(1,3,5)^.

RO had a strong negative correlation with CPP, a moderate negative correlation with CPPS, and a weak positive correlation with H1-H2. CPP and CPPS are considered sensitive measures to the presence of breathiness in voice emission. Computer models^(14)^ have demonstrated that CPP is sensitive to the various factors underlying the hearing perception of breathiness, such as distancing in vocal processes, changes on the surface of the free edges of the vocal folds, and decreased constriction in the epilaryngeal area.

On the other hand, although H1-H2 is indicated as a measure sensitive to the presence of breathiness, experimental conditions verified that the H1-H2 measure was only sensitive to the presence of breathiness originating in the distancing of vocal processes when all other structural and kinematic variables of the vocal folds remained constant^(14)^. Nonetheless, changes in the free edge of the vocal folds and constrictions implemented in the vocal tract may have a greater influence on H1-H2 values and the association between this measure and the perception of breathiness^(14,15)^.

RO demonstrated a moderate negative correlation with CPPS and a weak negative correlation with CPP. Roughness and breathiness obviously overlap in dysphonic voices, as previously mentioned. Such overlapping increases in more intense vocal deviations, which explains the moderate to strong negative correlation between CPP and CPPS and BR and the weak to moderate correlation between these measures and RO.

Lastly, there was a weak positive correlation between ST and spectral decrease. Even though the spectral decrease is influenced by structural and kinematic characteristics of the vocal folds, subglottal pressure seems to be the main determinant of such values^(32)^. Increased subglottal pressure, in turn, is related to the hearing perception of phonatory strain^(6)^, which may explain the correlation between spectral decrease and ST found in this research.

Although H1-H2 measures and spectral decrease are considered potential indicators of the presence of strain in the voice quality^(26,27)^, they were not found in this study. The etiology of strain in the voice quality is evidently multifactorial, and the various factors influence differently the magnitude of changes in cepstral and spectral measures. For instance, the vertical thickness of the vocal folds has a greater effect on determining the spectral contour and the energy of the upper harmonics than longitudinal (anteroposterior) strain in the vocal folds. In this sense, the absence of correlation between ST and CPP, CPPS, and H1-H2 does not indicate that such measures are not useful in clinical assessment but restates that acoustic parameters must be cautiously interpreted and associated with other clinical information obtained from multidimensional voice assessment.

The findings in this research may reinforce that the voice production process occurs in the time domain while hearing perception of the voice quality is strongly influenced by spectral information. Thus, different glottal/supraglottal adjustments and laryngeal conditions may produce the same vocal output. Likewise, greater changes in these adjustments may not change the vocal output^(10,33)^. The various adjustments interact nonlinearly to make an auditory impression related to voice quality. Moreover, the data found in this research seem to reinforce the importance of acoustic analysis as an additional tool to estimate structural and kinematic aspects underlying voice production. They must be cautiously used to understand dysphonic patients’ voice problems.

This study categorized the groups in terms of the presence/absence of laryngeal lesions. This method was necessary to ensure the internal and external validity of the study concerning its objectives. Despite the differences found in the investigated measures between the groups with and without laryngeal lesions, the conscious and pragmatic use of acoustic measures is recommended in the clinical assessment process to confirm diagnoses or monitor patients with voice complaints. Acoustic measures mainly help speech-language-hearing therapists understand the patient’s voice quality and underlying physiological, aerodynamic, and biomechanical factors, which cannot be directly and routinely accessed in clinical practice without using high-cost technology (such as aerodynamic assessment systems).

The results of this research were limited to women with behavioral dysphonia, with or without laryngeal lesions (specifically, vocal nodules) in the vocal folds. This must be considered when interpreting and transferring these findings to everyday clinical conditions. This study did not recruit vocally healthy individuals, which is a limitation to be addressed in future investigations. Furthermore, normative H1-H2 data must be developed for vocally healthy Brazilian Portuguese speakers of both sexes.

One of the limitations of this study was that acoustic measures were taken only from the sustained vowel samples, not including linked speech. The discriminative power of cepstral and spectral measures to distinguish voice samples of dysphonic and non-dysphonic individuals may be influenced by speech tasks^(23,24)^. Moreover, APE in this research approached samples combining sustained vowels and linked speech (counting numbers), whereas acoustic parameters were extracted only from the vowel. Hence, the correlation analysis between APE and acoustic measures may have been influenced by such methods. Thus, further studies may investigate whether the correlation strength changes when the investigation considers APE and acoustic measure data obtained from linked speech tasks.

## CONCLUSION

BDWL women had higher H1-H2 values and lower CPP and CPPS values than BDWOL women. More deviant voices had lower CPP and CPPS values than less deviant voices. Breathy voices had lower CPP and CPPS values and higher H1-H2 values than rough ones.

GH had a moderate negative correlation with CPP and CPPS. RO had a weak negative correlation with CPP and a moderate negative correlation with CPPS. BR had a strong negative correlation with CPP, a moderate negative correlation with CPPS, and a weak positive correlation with H1-H2. ST had a weak positive correlation with spectral decrease.
