# Supplementary material for: Spectral and cepstral measurements in women with behavioral dysphonia
Source: Codas. 2023 Nov 10;36(1):e20220327. doi: 10.1590/2317-1782/20232022327en (PMC10715576; doi:10.1590/2317-1782/20232022327en)
Supplement: Translation

# Translation: Medidas espectrais e cepstrais em mulheres com disfonia comportamental

**DOI:** 10.1590/2317-1782/20232022327pt

**Authors:** Gabriela Marques Paiva, Priscila Oliveira Costa Silva, Layla Jamilly Andrade da Silva, Kézia Alves Nascimento, Ana Beatriz da Veiga e Silva, Samuel Ribeiro de Abreu, Anna Alice Figueiredo de Almeida, Leonardo Wanderley Lopes

**Affiliations:** 1 Universidade Federal da Paraíba - UFPB - João Pessoa (PB), Brasil.

**Keywords:** Voz, Qualidade Vocal, Acústica

## Abstract

**Objetivo:**

Investigar se existem diferenças nas medidas acústicas cepstrais e espectrais entre mulheres com disfonia comportamental com e sem lesão laríngea, bem como verificar se existe correlação entre tais medidas e o julgamento perceptivo-auditivo da qualidade vocal.

**Método:**

Participaram 78 mulheres com disfonia comportamental sem lesão laríngea (DCSL) e 68 com disfonia comportamental com lesão laríngea (nódulos vocais) (DCCL). Foram extraídas as medidas CPP (cepstral peak prominence), CPPS (cepstral peak prominence smoothed), declínio espectral e H1-H2 (diferença entre a amplitude do primeiro e do segundo harmônico), assim como o julgamento perceptivo-auditivo (JPA) do grau geral de desvio vocal (GG), graus de rugosidade (GR), de soprosidade (GS) e de tensão (GT).

**Resultados:**

Mulheres com DCCL apresentaram maiores valores de H1-H2 e menores valores no CPP e CPPS, em relação às mulheres com DCSL. As vozes mais desviadas apresentaram menores valores do CPP e CPPS. As vozes soprosas apresentaram menores valores de CPP e CPPS, assim como maior valor de H1-H2 em relação às vozes rugosas. Houve correlação negativa fraca entre o CPP e o GR, negativa moderada com o GG e negativa forte com o GS. O CPPS apresentou correlação negativa moderada com o GG, GR e GS. A medida H1-H2 apresentou correlação positiva fraca com o GS. Houve correlação positiva fraca entre o declínio espectral e o GT.

**Conclusão:**

As medidas acústicas H1-H2, CPP e CPPS apresentam diferenças entre mulheres com DCSL e DCCL. Além disso, há correlação entre as medidas cepstrais e espectrais e os diferentes parâmetros do JPA.

## INTRODUÇÃO

A análise acústica é uma ferramenta capaz de caracterizar o sinal vocal de maneira quantitativa ou qualitativa e, assim, produzir estimativas acerca dos aspectos fisiológicos subjacentes à produção vocal^(1)^. Na perspectiva clínica, espera-se que cada medida acústica seja sensível à determinadas variações aerodinâmicas e biomecânicas da produção vocal, assim como associadas aos parâmetros do julgamento perceptivo-auditivo (JPA).

Entre as medidas acústicas, há um maior número de pesquisas e aplicações utilizando as medidas baseadas em estatísticas e perturbações de curto termo da frequência de oscilação (fo), perturbações de curto termo da amplitude, assim como perturbações da forma da onda^(2)^. No entanto, tais medidas apresentam restrições importantes relacionadas à confiabilidade e reprodutibilidade, principalmente na avaliação de vozes com desvios mais intensos^(2)^. Nesse sentido, as pesquisas^(3,4)^ e consenso^(5)^ recentes têm apoiado o uso das medidas espectrais, sejam aquelas baseadas na transformada de Fourier/espectro LPC (*Linear Predictive Coding*) ou baseadas no cepstro, para a avaliação acústica de vozes disfônicas. Entre tais medidas, a proeminência do pico cepstral (CPP - *Cepstral Peak Prominence*) e sua versão suavizada (CPPS - *Cepstral Peak Prominence Smoothed*) têm sido indicadas como medidas confiáveis para avaliação de vozes disfônicas, independentemente da intensidade do desvio vocal^(1,3-6)^.

O CPP e o CPPS evidenciam em que medida os harmônicos advindos da fo são individualizados e se destacam em relação ao nível de ruído presente no sinal^(3)^. Sinais com maior regularidade e menor quantidade de ruído apresentam maior definição e amplitude do pico cepstral dominante. O CPP e o CPPS têm duas vantagens principais em relação às medidas tradicionais de perturbação e ruído: independem da estimativa de fo para serem obtidas e podem ser extraídas tanto da vogal sustentada quanto da fala encadeada. Além disso, os valores do CPPS foram capazes de discriminar entre falantes do português brasileiro com e sem desvio vocal; diferenciar entre vozes predominantemente rugosas, soprosas e tensas; assim como apresentou correlação negativa forte com o grau geral de desvio vocal (GG) e grau de soprosidade (GS), correlação negativa moderada com o grau de rugosidade (GR) e correlação negativa fraca com o grau de tensão (GT)^(6)^.

No campo das medidas espectrais, as medidas relacionadas ao declínio espectral, que incluem parâmetros que refletem o espectro da energia harmônica, comparando as diferenças em decibel (dB) entre as amplitudes relativas de dois harmônicos, estão entre os principais preditores do hiperfuncionamento vocal ou do componente de soprosidade relacionados à produção vocal^(7,8)^. A diferença de amplitude entre o primeiro e o segundo harmônicos (H1-H2) e a caracterização do declínio espectral expresso em decibel (dB) estão entre as principais medidas recomendadas para utilização em contexto clínico^(7)^. Em uma revisão sistemática com metanálise, o H1-H2 esteve entre as principais medidas para predizer a presença e a intensidade da soprosidade na emissão vocal^(4)^.

H1-H2 é uma medida relacionada à inclinação espectral, à inclinação do pulso de fluxo de ar glotal, ao quociente de abertura das pregas vocais, à espessura da borda livre das pregas vocais e à assimetria de vibração entre as pregas vocais^(9,10)^. Além disso, essa medida está associada com os componentes de soprosidade e tensão. H1-H2 é capaz de fornecer *insights* sobre os mecanismos fisiológicos subjacentes à produção vocal, com potencial uso na avaliação clínica de indivíduos disfônicos.

De maneira geral, pacientes com lesão fonotraumática nas pregas vocais têm valores mais elevados de H1-H2 em comparação a indivíduos vocalmente saudáveis^(8)^. Maiores valores de H1-H2 estão associados às seguintes características: contato reduzido entre as pregas vocais, padrão vibratório com fechamento menos abrupto entre as pregas vocais, maior duração da fase aberta dos ciclos glóticos, maior constrição/estreitamento no trato vocal e qualidade vocal soprosa^(8,9,11,12)^. Por sua vez, menores valores de H1-H2 são compatíveis com maior fechamento glótico ou fechamento glótico abrupto das pregas vocais, com maior componente de tensão fonatória na qualidade vocal^(8)^, aumento da tensão longitudinal (anteroposterior) das pregas vocais^(13)^, aumento da espessura vertical da borda livre das pregas vocais^(13)^.

H1-H2 é uma medida com grande variabilidade na comparação intersujeitos^(10)^. Dessa forma, a medida H1-H2 necessita ser investigada em diferentes condições laríngeas e de desvio da qualidade vocal para compreensão da sua utilidade no contexto clínico. Além disso, o CPP/CPPS, H1-H2 e o declínio espectral têm sido consideradas medidas complementares para avaliação do desvio vocal e, consequentemente, para estimar as mudanças estruturais e cinemáticas das pregas vocais subjacentes ao processo de produção vocal^(14,15)^.

As disfonias comportamentais correspondem a 65% dos casos encontrados na clínica vocal^(16)^, sendo que 10 a 40% desses casos podem ocorrer na ausência de alteração estrutural na laringe^(17)^. Pacientes com disfonia comportamental, independentemente da presença de lesão fonotraumática, apresentam aumento da atividade muscular, esforço ou tensão excessiva relacionada à musculatura intrínseca e/ou extrínseca da laringe durante a produção vocal^(18)^. Por sua vez, a presença de uma lesão tecidual associada ao fonotrauma, como os nódulos, pólipos e edema nas pregas vocais, têm potencial de modificar as características vibratórias e cinemáticas relacionadas à produção vocal^(18)^. Dessa forma, a prevalência desses quadros justifica a importância da identificação de medidas capazes de caracterizar e monitorar a produção vocal desses pacientes, com potencial aplicação na prática clínica.

Sendo assim, considerando-se a relevância dessas medidas no processo de avaliação clínica da voz e a escassez de estudos com falantes do português brasileiro (PB), o objetivo dessa pesquisa foi investigar se existem diferenças nas medidas acústicas cepstrais (CPP e CPPS) e espectrais (H1-H2 e declínio espectral) entre mulheres com disfonia comportamental com (DCCL) e sem lesão laríngea (DCSL), bem como verificar se existe correlação entre tais medidas e o JPA da qualidade vocal.

## MÉTODO

### Desenho do estudo

Este é um estudo transversal e descritivo, avaliado e aprovado pelo Comitê de Ética em Pesquisa da instituição de origem com parecer de nº 2.677.777/2018. Todas as participantes assinaram o Termo de Consentimento Livre e Esclarecido (TCLE), concedendo anuência para participação na pesquisa.

### Amostra

A amostra desta pesquisa foi composta por 146 mulheres disfônicas com média de idade de 33,10±11,26 anos, avaliadas no laboratório de voz da Instituição de origem em momento anterior à realização da terapia vocal. Entre as participantes, 78 receberam o diagnóstico de disfonia comportamental sem lesão laríngea e 68 foram diagnosticadas com disfonia comportamental com lesão laríngea (nódulos vocais).

Foram considerados os seguintes critérios de elegibilidade para a seleção das participantes: ser do gênero feminino, considerando-se que as medidas acústicas investigadas no presente estudo são influenciadas pela variável gênero^(19)^; idade superior a 18 anos para evitar período da muda vocal; apresentar queixa vocal, respondendo positivamente à pergunta: “Você considera que tem um problema de voz atualmente?”; diagnóstico de disfonia comportamental (com nódulos vocais ou sem lesão laríngea), confirmado por meio de exame visual laríngeo realizado por médico otorrinolaringologista.

Os critérios para exclusão das participantes consideraram: mulheres com uso profissional da voz ou que tivessem se submetido à cirurgia de cabeça e pescoço (incluindo os casos de microcirurgia de laringe) ou terapia vocal prévia. Além disso, foram estabelecidos critérios de exclusão relacionados à qualidade dos sinais de voz coletados. Seriam excluídos do estudo as participantes cujos sinais de voz apresentassem a relação sinal-ruído (*Signal to noise Ratio* - SNR) abaixo de 30dB NPS^(5)^.

Na Figura 1 podem ser conferidas as informações relacionadas à seleção da amostra baseada nos critérios de inclusão e exclusão.

**Figura 1:** Fluxograma dos participantes da pesquisa, de acordo com os critérios de elegibilidade

As participantes foram alocadas em um dos seguintes grupos:

- Disfonia comportamental sem lesão laríngea (DCSL) - incluindo participantes cujo fator etiológico da disfonia está associado ao comportamento vocal, com autorreferência de problema de voz. Nenhuma das participantes desse grupo apresentava alteração estrutural na laringe, sendo que 25 receberam diagnóstico otorrinolaringológico de fenda triangular médio-posterior, 11 participantes com diagnóstico de constrição supraglótica e 42 participantes com laudo de laringe normal.
- Disfonia comportamental com lesão laríngea (DCCL) - mulheres com alteração estrutural nas pregas vocais cujo fator etiológico da disfonia está associado ao comportamento vocal, com autorreferência de problema de voz. As 68 participantes desse grupo receberam diagnóstico otorrinolaringológico de nódulos vocais e presença de fenda glótica. Nesta pesquisa, preconizou-se o diagnóstico de nódulos vocais, considerando-se que a principal lesão laríngea associada às disfonias comportamentais^(16)^.

### Procedimento de coleta dos dados

A coleta de dados desta pesquisa foi realizada no Laboratório de Voz de uma Instituição de Ensino Superior. Inicialmente, as participantes são avaliadas por meio de uma ficha, com abordagem aos dados pessoais e presença de queixa vocal. Em seguida foi realizada a gravação das amostras vocais.

Para a coleta de voz, utilizou-se o *software* Fonoview, versão 4.5, da CTS Informática, *desktop* Dell all-in-one, microfone cardioide unidirecional, da marca Sennheiser, modelo E-835, localizado em um pedestal e acoplado a um pré-amplificador Behringer, modelo U-Phoria UMC 204. As vozes foram coletadas em cabine de gravação com tratamento acústico e ruído inferior a 50 dB NPS, com taxa de amostragem de 44000 Hz, com 16 bits por amostra e distância de 10 cm entre o microfone e a boca da participante.

Para a coleta das vozes, as participantes estavam em pé, situando-se o pedestal a sua frente, de acordo com a distância preconizada entre a boca e o microfone. A participante recebeu as instruções sobre a coleta de voz e, logo em seguida, procedeu-se com o registro. Durante a gravação, a participante foi solicitada a emitir a vogal /a/ sustentada com duração mínima de cinco segundos, em frequência e intensidade autorreferida como habitual, assim como a contagem de números de 1 a 10.

Durante o registro das vozes, os sinais foram monitorados visualmente no display do *software Fonoview*, observando-se a duração dos sinais (atingindo uma duração mínima de 5s) e a presença de *peak clipping*. Dessa forma, nos casos de duração menor que 5s ou de *peak clipping,* a participante era convidada a refazer a gravação para obtenção dos critérios estabelecidos.

Após a sessão de coleta de dados e gravação das amostras vocais, as participantes foram encaminhadas para o exame médico otorrinolaringológico, que incluía o exame visual da laringe, que consistiu na telelaringoscopia com endoscópio rígido. Todos os exames foram realizados no mesmo serviço de referência e os participantes recebiam laudo por escrito com o diagnóstico laríngeo. O resultado do exame foi utilizado como critério de seleção das participantes da pesquisa.

Na sequência, antes do JPA e da extração das medidas acústicas, foi utilizado o *script VoxMore* do *software* de acesso aberto Praat (Paul Boersma e David Weenink, University of Amsterdam, Holanda), versão 5.3.84 para obtenção do SNR dos sinais vocais. Todas as amostras das 146 participantes selecionadas cumpriram os critérios de qualidade do sinal previamente definidos^(5)^, com SNR de 45,37± 3.1 dB NPS.

As amostras da vogal e contagem foram apresentadas a um fonoaudiólogo especialista em voz, com mais de 20 anos de experiência em avaliação e tratamento dos distúrbios da voz. A sessão de JPA ocorreu em um ambiente silencioso, com fones de ouvido acoplados a um laptop e volume confortável autorreferido pelo avaliador. Para o JPA, o fonoaudiólogo escutou as amostras vocais (contagem e vogal sustentada) de forma encadeada, com intervalo de um segundo entre elas. Na sequência, deveria proceder com a marcação em uma Escala Analógico-Visual (EAV) com intervalo entre 0 e 100 mm, julgando o GG, GR, GS e GT. A marcação mais próxima do 0 representa menor desvio da qualidade vocal e, quanto mais próxima do 100, mais desviada é a qualidade vocal^(20)^.

Ao final da sessão de JPA, 20% (n=30) das amostras vocais foram repetidas aleatoriamente, para a análise da confiabilidade intra-avaliador através do Coeficiente Kappa de Cohen. Tal juiz obteve um coeficiente Kappa de 0,83, indicando boa concordância.

Os valores do ponto de corte da EAV^(20)^ foram utilizados para classificar as vozes quanto à presença de desvio vocal e ao GG. Os sinais avaliados com valores ≤ 35,5 mm foram considerados como qualidade vocal adaptada ou com VNVQ (variabilidade normal da qualidade vocal), enquanto as vozes com valores > 35,5 mm foram classificadas como desviadas. Posteriormente foi realizada uma correspondência da EAV para a escala numérica (EN), sendo o grau 1 (0-35,5 mm) relacionado à variabilidade normal da qualidade vocal (VNQV), grau 2 (35,6-50,5 mm) desvio leve a moderado, grau 3 (50,6-90,5 mm) desvio moderado e grau 4 (90,6-100 mm) desvio intenso^(20)^. Após o resultado do JPA, observou-se que 11 mulheres apresentaram qualidade vocal adaptada, 74 mulheres com desvio vocal de leve a moderado, e 61 com desvio vocal moderado. Nenhuma participante selecionada para o presente estudo apresentou desvio vocal intenso. Para as 74 mulheres com desvio vocal, o maior valor na EAV para o GR, GS e GT foi utilizado para classificação do predomínio da qualidade vocal desviada como rugosa, soprosa ou tensa.

Optou-se pela manutenção das 11 participantes com qualidade vocal adaptada no presente estudo, considerando-se que a confirmação diagnóstica da disfonia é multidimensional e que nos quadros comportamentais podem ocorrer múltiplas interações entre parâmetros fisiológicos, acústicos, perceptivo-auditivos e de autopercepção do problema de voz^(18)^. Desse modo, mulheres com disfonia comportamental podem apresentar qualidade vocal adaptada, mesmo com a referência de queixas/sintomas vocais e presença de alteração estrutural na laringe. Na amostra desse estudo, das 11 mulheres com qualidade vocal adaptada, oito estavam no grupo DCSL e apenas três estavam alocadas no grupo DCCL.

Além disso, deve-se destacar que o estudo de referência^(20)^ utilizado na realidade brasileira para determinação dos valores de corte da EAV utilizou como tarefa de fala apenas a contagem de 1 a 10 (fala encadeada). Embora tal fato possa se constituir em uma limitação do presente estudo, optou-se pela utilização dos valores de corte propostos por Yamasaki et al.^(20)^ por utilizar apenas os quatro graus de desvio considerados internacionalmente (adaptada ou VNQV, leve a moderado, moderado e intenso) e ser a principal referência brasileira para os valores de corte utilizados nessa classificação. Além disso, a tarefa de contagem de 1 a 10 foi incluída (juntamente com a vogal sustentada) durante o procedimento de JPA.

Na sequência, foi realizada a extração das medidas acústicas CPP, CPPS, declínio espectral e H1-H2 a partir da amostra da vogal /a/ sustentada. A amplitude do primeiro e do segundo harmônicos é influenciada pelo tipo de vogal coletada, principalmente quanto ao timbre (abertas *vs*. fechadas)^(21)^. Dessa forma, a vogal /a/ é a mais confiável para extração da medida H1-H2, visto que a frequência do primeiro formante é suficientemente alta para não interferir nos harmônicos nas faixas de frequências mais baixas. Além disso, há maior distintividade entre o primeiro e o segundo harmônicos nessa vogal^(8,21)^.

A extração das medidas foi realizada de maneira automática, utilizando-se o *script VoxMore* ^(22)^ no *software* Praat (v.5.3.84). Tal *script* possibilita a extração das medidas nos três segundos centrais da vogal sustentada. Especificamente, quanto à extração de H1-H2, o *script* utiliza a transformação discreta de Fourier, com janelas de 50 ms. O H1-H2 para cada janela será definido como a diferença, em dB, entre as amplitudes do primeiro e segundo harmônicos no espectro de frequência^(8)^. Para a extração do CPP foi utilizado um janelamento de 41 ms, de modo que o valor final do CPP para cada amostra correspondeu à média de todos os quadros de análise no intervalo de 3 s^(23,24)^. O CPPS foi obtido em janelas de 2 ms, com o valor final correspondente à média de todos os quadros de análise no intervalo de 3s^(3,25)^.

### Análise dos dados

Foi realizada análise estatística descritiva para todas as variáveis independentes analisadas, incluindo-se os valores de média e desvio padrão. Inicialmente, foi executado o teste de Shapiro-Wilk para verificar a curva de distribuição de normalidade das variáveis investigadas (GG, GR, GS, GT, CPP, CPPS, declínio espectral e H1-H2) na amostra do estudo. Dessa forma, considerando-se que houve distribuição normal de todas as variáveis citadas, o teste T para amostras independentes foi utilizado para comparação das médias das medidas acústicas e do JPA entre os grupos DCSL e DCCL. O teste ANOVA de um fator foi utilizado para comparação das medidas acústicas entre os grupos em função do GG e da qualidade vocal predominante, seguido pelo pós teste de Tukey quando houve diferença estatisticamente significante entre os grupos na ANOVA. O teste de correlação de Pearson foi utilizado para verificar se existia correlação entre os valores das medidas acústicas e os parâmetros do julgamento perceptivo-auditivo (GG, GR, GS e GT).

Os coeficientes de correlação foram utilizados para avaliar e quantificar o grau de relacionamento linear entre as variáveis acústicas (CPP, CPPS, declínio espectral e H1-H2) e perceptivo-auditivas (GG, GR, GS e GT), observando se as variáveis se modificam conjuntamente e em que grau. Para a classificação dos coeficientes de correlação, adotou-se, nesta pesquisa, que valores de 0,1 a 0,3 representam uma correlação fraca; entre 0,4 e 0,6 indicam correlação moderada; e acima de 0,6 é possível afirmar que o grau de correlação entre as variáveis é forte.

Todas as análises foram realizadas pelo *software Statistical Package for the Social Sciences* (SPSS) versão 2.0.O nível de significância adotado foi de 5%.

## RESULTADOS

Houve diferença em todas as medidas acústicas de H1-H2, CPP e CPPS entre os grupos de participantes investigados (Tabela 1). As mulheres com DCCL apresentaram maiores valores de H1-H2 (p=0,024), assim como menores valores no CPP (p=0,017) e no CPPS (p=0,004) em relação às mulheres com DCSL. Não houve diferenças nos valores do declínio espectral entre os grupos DCCL e DCSL (p=0,504).

**Tabela 1:** Comparação das médias dos valores das medidas acústicas e dos parâmetros do julgamento perceptivo-auditivo entre mulheres com disfonia comportamental, com e sem lesão laríngea

**MEDIDAS**	**GRUPO**		**Valor de p**
	**DCSL**	**DCCL**	
	**Média e DP**	**Média e DP**	
**GG**	43,03±8,18	55,83±12,10	<0,001*
**GR**	38,24±11,12	51,38±12,79	<0,001*
**GS**	38,01±13,66	48,19±17,39	<0,001*
**GT**	28,62±11,10	36,22±13,73	<0,001*
**CPP**	27,52±3,60	25,29±5,45	0,017*
**CPPS**	16,36±2,39	14,77±3,10	0,004*
**Declínio espectral**	-14,68±0,52	-15,33±0,63	0,504
**H1-H2**	1,65±5,31	3,64±5,16	0,024*

Teste T para amostras independentes

* Valores significativos - p-valor < 0,05

Legenda: **DP**= desvio-padrão; **GG** = grau geral de desvio vocal; **GR** = grau de rugosidade; **GS** = grau de soprosidade; **GT** = grau de tensão vocal; **H1-H2** = diferença de amplitude entre os dois primeiros harmônicos do espectro da voz; **CPP** = cepstral peak prominence; **CPPS** = cepstral peak prominence smoothed; **DCSL** = disfonia comportamental sem lesão laríngea; **DCCL** = disfonia comportamental com lesão laríngea

Observou-se diferença nos valores do CPP (p <0,001) e CPPS (p <0,001) em função do GG (Tabela 2). No pós teste (Teste de Tukey) para comparação dos grupos par a par, os valores do CPP apresentaram diferenças na comparação adaptada x leve a moderado (p= 0,004), adaptada x moderado (p <0,001), leve a moderado x moderado (p <0,001). Da mesma forma, os valores do CPPS apresentaram diferenças na comparação adaptada x leve a moderado (p= 0,005), adaptada x moderado (p <0,001), leve a moderado x moderado (p <0,001). De modo geral, as vozes mais desviadas apresentaram menores valores do CPP e CPPS. Não houve diferenças nos valores do declínio espectral (p=0,220) e H1-H2 (p=0,504) em função do GG (p=0,504).

**Tabela 2:** Comparação das médias dos valores das medidas acústicas e dos parâmetros do julgamento perceptivo-auditivo entre mulheres com disfonia comportamental, com e sem lesão laríngea, em função do grau geral do desvio vocal

**MEDIDAS**	**GRAU DO DESVIO VOCAL**			**Valor de p**
	**Adaptada**	**Leve a moderado**	**Moderado**	
	**Média e DP**	**Média e DP**	**Média e DP**	
**CPP**	31,86±3,25	27,52±3,39	24,25±5,04	<0,001*
**CPPS**	18,98±2,22	16,33±2,32	14,15±2,71	<0,001*
**Declínio espectral**	-13,47±1,04	-15,23±0,57	-14,96±0,66	0,220
**H1-H2**	3,76±2,91	1,54±4,89	3,62±5,92	0,057

Teste Anova de um fator

* Valores significativos - p-valor < 0,05

Legenda: **DP** = desvio-padrão; **H1-H2** = diferença de amplitude entre os dois primeiros harmônicos do espectro da voz; **CPP** = *cepstral peak prominence*; **CPPS** = *cepstral peak prominence smoothed*

Houve diferença nas medidas de CPP (p <0,001), CPPS (p <0,001) e H1-H2 (p 0,022) em função da qualidade vocal predominante (Tabela 3). No pós teste (Teste de Tukey) para comparação dos grupos par a par, observou-se que os valores do CPP (p <0,001), CPPS (p <0,001) e H1-H2 (0,018) apresentaram diferenças apenas na comparação entre a qualidade vocal rugosa e soprosa. As vozes soprosas apresentaram menores valores de CPP e CPPS, assim como maior valor de H1-H2 em relação às vozes rugosas. Não houve diferenças nos valores do declínio espectral (p=0,671) em função da qualidade vocal predominante.

**Tabela 3:** Comparação das médias dos valores das medidas acústicas e dos parâmetros do julgamento perceptivo-auditivo entre mulheres com disfonia comportamental, com e sem lesão laríngea, em função da qualidade vocal predominante

**MEDIDAS**	**QUALIDADE VOCAL PREDOMINANTE**			**Valor de p**
	**Rugosa**	**Soprosa**	**Tensa**	
	**Média e DP**	**Média e DP**	**Média e DP**	
**CPP**	27,53±4,13	24,61±4,49	28,38±1,58	<0,001*
**CPPS**	16,14±2,45	14,57±2,78	16,80±1,47	<0,001*
**Declínio espectral**	-15,49±0,64	-15,30±0,54	-5,84±2,75	0,671
**H1-H2**	0,98±5,44	3,68±5,23	4,35±5,14	0,022*

Teste Anova de um fator

* Valores significativos - p-valor < 0,05

Legenda: **DP** = desvio-padrão; **H1-H2** = diferença de amplitude entre os dois primeiros harmônicos do espectro da voz; **CPP** = *cepstral peak prominence*; **CPPS** = *cepstral peak prominence smoothed*

Por fim, o CPP demonstrou correlação negativa fraca com o GR (<0,001), negativa moderada com o GG (<0,001) e negativa forte com o GS (<0,001) (Tabela 4). O CPPS apresentou correlação negativa moderada com o GG (<0,001), GR (<0,001) e GS (<0,001). A medida H1-H2 apresentou apenas correlação positiva fraca com o GS (p=0,013). Houve correlação positiva fraca entre o declínio espectral e o GT (p=0,003).

**Tabela 4:** Correlação entre a intensidade do desvio vocal nos diferentes parâmetros perceptivo-auditivos e as medidas acústicas

**MEDIDA ACÚSTICA**	**VARIÁVEIS**							
	**GG**		**GR**		**GS**		**GT**	
	**Corr**	**p-valor**	**Corr**	**p-valor**	**Corr**	**p-valor**	**Corr**	**p-valor**
**CPP**	-0,45	<0,001*	-0,31	<0,001*	-0,60	<0,001*	-0,03	0,693
**CPPS**	-0,52	<0,001*	-0,40	<0,001*	-0,58	<0,001*	-0,10	0,210
**Declínio espectral**	0,07	0,933	-0,021	0,802	-0,086	0,303	0,24	0,003*
**H1-H2**	-0,12	0,208	-0,15	0,783	0,08	0,013*	-0,08	0,313

Teste de Correlação de Pearson

* Valores significativos (p≤0,05)

Legenda: **GG** = grau geral de desvio vocal; **GR** = grau de rugosidade; **GS** = grau de soprosidade; **GT** = grau de tensão vocal; **Corr** = coeficiente de correlação; **H1-H2** = diferença de amplitude entre os dois primeiros harmônicos do espectro da voz; **CPP** = *cepstral peak prominence*; **CPPS** = *cepstral peak prominence smoothed*

## DISCUSSÃO

As medidas acústicas espectrais/cepstrais têm despontado como as mais promissoras para avaliação e monitoramento de vozes disfônicas. Elas demonstraram ser sensíveis a diferentes componentes do JPA (tais como o GG, GS e GT)^(6)^ e às características cinemáticas e vibratórias associadas à produção vocal normofônica e disfônica^(14)^.

Nesta pesquisa, as mulheres com DCCL apresentaram maiores valores na H1-H2, assim como menores valores no CPP e no CPPS em relação às mulheres com DCSL. Embora não haja dados normativos de H1-H2 para mulheres falantes do português brasileiro, há uma variabilidade de 3,3-8,4 dB nos valores dessa medida para falantes do inglês^(21)^. No presente estudo, as mulheres DCSL e DCCL apresentaram valores de 1,65±5,31 e 3,64±5,16, respectivamente. Tais valores podem ser sugestivos de maior fechamento glótico ou fechamento glótico abrupto das pregas vocais, com maior componente de tensão fonatória na qualidade vocal^(8)^, aumento da tensão longitudinal (anteroposterior) das pregas vocais^(13)^ e aumento da espessura vertical da borda livre das pregas vocais^(13)^ em mulheres disfônicas.

Por sua vez, os maiores valores de H1-H2 em mulheres com DCCL em comparação às mulheres com DCSL podem indicar contato mais reduzido entre as pregas vocais, padrão vibratório com fechamento menos abrupto entre as pregas vocais, maior duração da fase aberta dos ciclos glóticos, maior constrição/estreitamento no trato vocal e maior componente de soprosidade^(8,9,11,12)^.

Pacientes com DCCL tendem a apresentar fenda glótica, o que pode justificar a presença de maior componente de soprosidade e os valores mais elevados de H1-H2^(8,26,27)^. Provavelmente, o grupo DCSL pode ter apresentado maior componente hiperfuncional e maior grau de fechamento glótico ou fechamento glótico abrupto, justificando os valores mais reduzidos de H1-H2.

Estudos anteriores^(8,9,26)^ compararam pacientes com lesão fonotraumática e indivíduos vocalmente saudáveis, observando que os valores de H1-H2 eram menores no grupo com lesão. No entanto, os dados da presente pesquisa podem indicar que, quando comparadas pacientes com DCCL e DCSL, os valores de H1-H2 demonstraram ser maiores nas pacientes com DCCL. Dessa forma, parece haver um *continuum* na medida H1-H2, com maiores valores em pessoas vocalmente saudáveis, redução de H1-H2 nos quadros de DCCL e menores valores na DCSL. Esse achado pode ser compatível com fechamento glótico mais intenso em pessoas com disfonia comportamental em relação àquelas vocalmente saudáveis, o que se reflete em menores valores de H1-H2.

Além disso, há correlação positiva moderada entre a assimetria de vibração das pregas vocais e os valores de H1-H2^(28)^. Sendo assim, a presença dos nódulos vocais no grupo DCCL pode ocasionar maior assimetria vibratória entre as pregas vocais e justificar os maiores valores de H1-H2 nesse grupo.

Quanto à comparação das medidas acústicas investigadas em função do GG, apenas o CPP e o CPPS apresentaram diferenças, com menores valores em pacientes com vozes mais desviadas. O CPPS e o CPPS têm sido recomendados como medidas úteis para monitorar o desvio da qualidade vocal durante o tratamento oferecido ao paciente^(3,6)^. Tais medidas são sensíveis à periodicidade do sinal e à amplitude da energia harmônica, portanto, recomendadas como a principal medida para monitorar o GG^(5)^.

Os resultados com relação à medida H1-H2 em função do GG são conflitantes na literatura. Não foram observadas modificações de H1-H2 consistentes na comparação entre vozes com maior ou menor GG^(29)^. Vozes com desvios intensos apresentaram valores semelhantes às vozes sem desvio^(29)^. Além disso, os valores de H1-H2 não reduziram após a terapia vocal em pacientes disfônicos, mesmo com a redução do GG e melhora nos parâmetros videoestroboscópicos da laringe^(30)^. Dessa forma, H1-H2 parece ser complementar às outras medidas como o CPP e CPPS, e útil para compreender a qualidade vocal predominante e a fisiologia subjacente ao processo de produção vocal.

Mulheres disfônicas com vozes predominantemente soprosas apresentaram menores valores de CPP e CPPS, assim como maior valor de H1-H2 em relação às mulheres disfônicas com vozes predominantemente rugosas. A diferenciação ente vozes disfônicas rugosas e soprosas a partir de medidas acústicas é desafiadora, uma vez que há sobreposição desses dois componentes em um mesmo sinal vocal desviado.

Existem três indicadores acústicos da presença de soprosidade na produção vocal: periodicidade do sinal, declínio espectral e amplitude do primeiro harmônico^(24)^. Entre esses indicadores, a periodicidade do sinal é capaz de explicar em 80% a variabilidade na percepção do grau de soprosidade presente em uma voz^(24)^. O CPP e o CPPS são medidas relacionadas à periodicidade do sinal, o que justificaria as diferenças encontradas nessas medidas entre as vozes rugosas e soprosas na presente pesquisa, com menores valores nas vozes soprosas.

Vozes soprosas tendem a apresentar maior amplitude do primeiro harmônico, com harmônicos superiores relativamente fracos, o que justifica os maiores valores de H1-H2 em vozes soprosas quando comparados às vozes rugosas^(24)^. Dessa forma, considerando-se que essas três medidas são sensíveis à presença do componente de soprosidade na produção vocal^(31)^, pode-se compreender a diferença de valores entre vozes predominantemente rugosas e soprosas.

Na presente pesquisa, o GG apresentou correlação negativa moderada com o CPP e CPPS. Tal achado suporta a indicação atual do CPP e CPPS como principais medidas para identificar a presença e a intensidade do desvio vocal em vozes disfônicas^(1,3,5)^.

O GS apresentou correlação negativa forte com o CPP, negativa moderada com o CPPS e positiva fraca com H1-H2. O CPP e CPPS são medidas consideradas sensíveis à presença do componente de soprosidade presente na emissão vocal. Modelos computacionais^(14)^ têm demonstrado que o CPP é sensível aos diferentes fatores subjacentes à percepção auditiva de soprosidade, tais como o afastamento dos processos vocais, as modificações na superfície da borda livre das pregas vocais e a redução da constrição na área epilaríngea.

Por outro lado, embora H1-H2 seja indicada como uma medida sensível à presença do componente de soprosidade, foi observado em condições experimentais que a medida H1-H2 só foi sensível à presença de soprosidade advinda de afastamento dos processos vocais, quando todas as outras variáveis estruturais e cinemáticas das pregas vocais eram mantidas constantes^(14)^. Por outro lado, as modificações de borda livre das pregas vocais e as constrições implementadas no trato vocal podem ter mais influência nos valores de H1-H2, e na associação entre essa medida e a percepção de soprosidade^(14,15)^.

O GR demonstrou correlação negativa moderada com o CPPS e negativa fraca com o CPP. Obviamente, em vozes disfônicas há uma sobreposição dos componentes de rugosidade e soprosidade, conforme citado anteriormente. Essa sobreposição é maior em função da intensidade do desvio vocal. Dessa forma, pode-se compreender o achado de correlação negativa de moderada a forte do CPP e CPPS com o GS, e a correlação de fraca a moderada dessas medidas com o GR.

Por fim, observou-se correlação positiva fraca entre o GT e o declínio espectral. Embora o declínio espectral seja influenciado por características estruturais e cinemáticas das pregas vocais, a pressão subglótica parece ser o principal fator determinante nos valores dessa medida^(32)^. Por sua vez, o aumento da pressão subglótica tem relação com a percepção auditiva de tensão fonatória^(6)^, o que pode fundamentar a correlação encontrada entre o declínio espectral e o GT na presente pesquisa.

Pode-se destacar que, embora as medidas H1-H2 e o declínio espectral sejam considerados como potenciais indicadores da presença de tensão na qualidade vocal^(26,27)^, tais achados não foram observados no presente estudo. Obviamente, a etiologia da tensão na qualidade vocal é multifatorial e os diferentes fatores influenciam de maneira diferente a magnitude das mudanças nas medidas cepstrais e espectrais. Por exemplo, a espessura vertical das pregas vocais tem maior efeito na determinação do contorno espectral e da energia nos harmônicos superiores, quando comparada à tensão longitudinal (anteroposterior) nas pregas vocais. Nesse sentido, a ausência de correlação entre o GT e o CPP, CPPS e H1-H2 não indica que tais medidas não sejam úteis para avaliação clínica, mas reforma que os parâmetros acústicos devem ser interpretados de maneira parcimoniosa e associadas às outras informações clínicas obtidas na avaliação multidimensional da voz.

Os achados desta pesquisa podem reforçar que o processo de produção vocal ocorre no domínio do tempo, enquanto a percepção auditiva da qualidade vocal é fortemente influenciada pela informação espectral. Dessa forma, diferentes ajustes glóticos/supraglóticos e diferentes condições laríngeas podem produzir o mesmo *output* vocal, assim como maiores mudanças nesses ajustes podem não ocasionar em mudanças no *output* vocal^(10,33)^. Os diferentes ajustes interagem entre si de maneira não linear para ocasionar uma impressão auditiva relacionada à qualidade vocal. Além disso, os dados encontrados parecem reforçar a importância da análise acústica como uma ferramenta adicional para estimar aspectos estruturais e cinemáticos subjacentes à produção vocal, que devem ser utilizados de maneira parcimoniosa para compreender o problema de voz do paciente disfônico.

No presente estudo, optou-se por categorizar os grupos em função da presença/ausência de lesão laríngea. Essa escolha metodológica era necessária para garantir a validade interna e externa do estudo em função dos objetivos propostos. Embora tenham sido observadas diferenças das medidas investigadas entre os grupos com e sem lesão laríngea, pode-se destacar que o uso consciente e pragmático das medidas acústicas é recomendado no processo de avaliação clínica para confirmação do diagnóstico ou monitoramento do paciente com queixa vocal. As medidas acústicas contribuem, principalmente, para a compreensão do fonoaudiólogo acerca da qualidade vocal do paciente e dos fatores fisiológicos, aerodinâmicos e biomecânicos subjacentes, os quais não podem ser acessados direta e rotineiramente na prática clínica sem o uso de tecnologias de alto custo (tais como os sistemas de avaliação aerodinâmica).

Os resultados desta pesquisa foram restritos a mulheres com disfonia comportamental, com (especificamente, nódulos vocais) e sem lesão nas pregas vocais, o que deve ser considerado no momento da interpretação e transferência desses achados para as condições clínicas cotidianas. No presente estudo, não foram recrutados indivíduos vocalmente saudáveis e essa é uma limitação a ser superada em investigações futuras. Além disso, há uma necessidade do desenvolvimento de dados normativos da medida H1-H2 para falantes do português brasileiro de ambos os gêneros e vocalmente saudáveis.

Uma das limitações do presente estudo está relacionada ao fato de que as medidas acústicas foram extraídas apenas para amostras da vogal sustentada e não incluíram trechos de fala encadeada. O poder discriminatório das medidas cepstrais e espectrais em diferenciar entre amostras vocais de indivíduos disfônicos e não disfônicos pode ser influenciado pela tarefa de fala^(23,24)^. Além disso, nesta pesquisa, o JPA foi realizado para uma amostra combinada da vogal sustentada e fala encadeada (contagem de números), enquanto a extração dos parâmetros acústicos baseou-se apenas na vogal. Dessa forma, a análise de correlação entre o JPA e as medidas acústicas pode ter sido influenciada por tais escolhas metodológicas. Nesse sentido, novos estudos podem investigar se há mudança na força de correlação quando investigada a correlação se considerados dados do JPA e medidas acústicas obtidas de tarefas de fala encadeada.

## CONCLUSÃO

As mulheres com DCCL apresentam maiores valores H1-H2, assim como menores valores no CPP e no CPPS em relação às mulheres com DCSL. As vozes mais desviadas apresentam menores valores de CPP e CPPS em relação às vozes menos desviadas. Vozes soprosas apresentam menores valores de CPP e CPPS, assim como maior valor de H1-H2 em relação às vozes rugosas.

O GG correlaciona-se de forma negativa moderada com o CPP e CPPS. O GR correlaciona-se de forma negativa fraca com o CPP e negativa moderada com o CPPS. O GS correlaciona-se de forma negativa forte com o CPP, negativa moderada com o CPPS e positiva fraca com H1-H2. O GT apresenta correlação positiva fraca com o declínio espectral.
